# Effects of Chlorogenic Acid-Rich *Eucommia ulmoides* Extract Supplementation During Late Gestation and Lactation on Maternal Health, Colostrum Composition, and Neonatal Intestinal Development

**DOI:** 10.3390/antiox15091198

**Published:** 2026-09-18

**Authors:** Yan Zhang, Hongda Pan, Anting Mao, Lianjiang Zhang, Hexuan Qu, Seongho Choi, Shuang Liang

**Affiliations:** 1 College of Animal Science and Technology, Jilin Agricultural Science and Technology College, Jilin 132109, China; 2 Department of Animals Sciences, College of Animal Sciences, Jilin University, Changchun 130062, China; 3 Department of Animal Science, Chungbuk National University, Cheongju 28644, Republic of Korea

**Keywords:** chlorogenic acid, maternal effects, fecal microbiota, colostrum metabolites, offspring development

## Abstract

Chlorogenic acid (CGA)-rich extract from *Eucommia ulmoides* (CAE) has antioxidant and anti-inflammatory properties; however, systematic investigations of its maternal effects on offspring development from late gestation through lactation are limited. This study investigated the effects of dietary CAE supplementation in sows during late gestation and lactation on maternal and offspring outcomes. Sows were randomly allocated to receive either a basal diet (CON) or the basal diet supplemented with 2000 mg/kg CAE. Compared with CON, CAE supplementation resulted in elevated maternal serum total antioxidant capacity (T-AOC) and catalase (CAT) activity, as well as immunoglobulin (IgA, IgG, and IgM) levels (*q* < 0.05). Placental villus capillary density and antioxidant enzyme activities, including superoxide dismutase (SOD) and CAT, were also greater (*q* < 0.05), accompanied by increased colostrum IgG concentration (*q* < 0.01). The CAE group exhibited greater litter-mean birth weight (*q* < 0.05). Offspring average daily gain (ADG) from birth to weaning was greater (*p* < 0.01), alongside higher serum T-AOC and CAT activity and immunoglobulin levels (*q* < 0.05) and enhanced intestinal morphological indices, including duodenal villus height and villus-to-crypt ratio (VH/CD) (*q* < 0.001). Fecal microbiota beta diversity differed between groups (ANOSIM: R = 0.687, *p* = 0.001), with phylum-level enrichment of *Firmicutes_A* and *Spirochaetota* and reduction in *Proteobacteria* and *Fusobacteriota*, alongside significant changes in multiple bacterial genera (*q* < 0.05). Exploratory metabolomic profiling of colostrum identified metabolite alterations associated with increased vitamin E acetate and 4-hydroxyacetophenone, altered amino acid and acylcarnitine profiles, and modified phospholipid signatures. These findings suggest that maternal CAE supplementation enhances maternal antioxidant status, alters placental microvascular morphology, modifies colostrum composition, and improves neonatal metrics and postnatal intestinal development. Because CAE supplementation spanned both late gestation and lactation, the relative contributions of gestational versus lactational exposure cannot be isolated; the functional significance of observed microbiota and metabolomic shifts requires further investigation through temporally partitioned experimental designs.

## 1. Introduction

Gestating sows have high metabolic rates, leading to a significant increase in reactive oxygen species (ROS) production. If excess ROS are not effectively scavenged, oxidative stress develops, which has been associated with impaired fetal development and reduced piglet survival. During late gestation, rapid fetal weight gain coincides with the placenta functioning as a highly active metabolic organ in which mitochondrial oxidative phosphorylation generates substantial amounts of ROS and becomes the primary source of gestational oxidative stress [1]. Furthermore, nutrient redirection toward the fetus depletes maternal antioxidant reserves, significantly reducing antioxidant capacity in late gestation, as evidenced by elevated oxidative stress biomarkers [2,3]. These synergistic factors have been shown to drive oxidative stress-mediated damage to placental vascular endothelial cells, reduce nutrient transport efficiency [4], and significantly increase the risk of intrauterine growth restriction (IUGR), characterized by reduced birth weight and an increased number of weak piglets [5,6]. During lactation, sows face particularly severe metabolic challenges because of the high energy and nutrient demands of milk production. This heightened metabolic load is associated with elevated systemic oxidative stress [7,8], which may impair mammary gland function and compromise the immunoglobulin concentrations in colostrum and milk, thereby potentially compromising passive immune protection and nutrient supply to piglets [9]. Consequently, late gestation and subsequent lactation represent critical high-risk stages for oxidative stress in sows.

Based on these established associations, oxidative stress-related pathways may influence intrauterine developmental programming and early growth trajectories in offspring through two proposed biological axes, the maternal–placental–fetal axis (during gestation) and the maternal–milk–piglet axis (during lactation), which could establish persistent maternal programming effects that shape offspring phenotype. The operational efficiency of these axes represents a critical determinant of offspring outcomes. In pigs, maternal antioxidant capacity has been closely associated with reproductive performance, neonatal piglet growth, and long-term health trajectories [10,11,12], reflecting its potential central role in maintaining axis functionality.

During gestation, placental angiogenesis serves as a critical determinant of the proposed maternal–placental–fetal axis, ensuring efficient nutrient and oxygen transfer. Existing evidence indicates that maternal oxidative stress compromises this process. Specifically, placentas from low-birth-weight piglets exhibit higher levels of oxidative damage, significantly reduced vascular density, and decreased expression of angiogenic markers [13]; compared with normal-birth-weight piglets, these placentas are more susceptible to vascular dysfunction when oxidative damage is aggravated [4]. During lactation, the proposed maternal–milk–piglet axis is functionally underpinned by colostrum quality for nutrient supply, immune modulation, and early microbial exposure. Colostrum is the sole source of energy and bioactive compounds [14,15] for neonatal piglets within the first hours after birth, and it also represents one of the earliest microbial exposures for piglets, with potential to influence gut microbiota colonization and gut health [16,17,18]. Studies have demonstrated that porcine colostrum is rich in bioactive components (such as sialyllactose, lactoferrin, immunoglobulins) and extracellular vesicles, which may contribute to immune regulation and promote beneficial bacterial colonization and maintain microbial homeostasis by modulating intestinal epithelial barrier function [19,20]. The gut microbiota plays a fundamental role in intestinal development, nutrient absorption, and immune maturation, thereby profoundly influencing growth performance [21,22,23]. Existing evidence suggests that maternal oxidative stress may alter colostrum composition [24], potentially reducing the concentrations of bioactive compounds that would support beneficial bacterial colonization and intestinal barrier integrity, and thereby compromise neonatal gut microbiota establishment and nutrient acquisition. If this hypothesis holds, nutritional intervention strategies that enhance maternal redox homeostasis represent an effective approach to improve placental angiogenesis during gestation, optimize colostrum composition, modulate offspring gut microbiota colonization, and promote intestinal development and microbial homeostasis in neonatal piglets during lactation, with potential benefits for perinatal outcomes.

The Dongliao black pig, an indigenous breed widely raised in northeast China, was selected as the experimental model. Compared with commercial lean-type genotypes, local pig breeds exhibit greater fat deposition capacity and distinct lipid metabolism patterns [25]. In sows, the high metabolic demands of late gestation and lactation are associated with marked systemic oxidative stress [8], and this association may be particularly pronounced in breeds with greater fat mobilization. Indeed, indigenous sows from northeast China, including the closely related Songliao black pig, display altered serum antioxidant profiles during gestation [26]. These characteristics render the Dongliao black pig a physiologically relevant model for evaluating whether maternal CAE supplementation alleviates oxidative stress and improves offspring outcomes via the maternal–offspring pathway. Furthermore, as an important regional genetic resource, this breed represents a valuable but understudied target for perinatal nutritional intervention; evidence generated from this breed may also inform nutritional management strategies for local pig breeds more broadly.

Given the established central role of oxidative stress in compromising placental and mammary function, nutritional strategies targeting maternal redox homeostasis represent a promising intervention. Among potential natural antioxidants, plant-derived polyphenols have garnered increasing attention due to their multifaceted bioactivities. *Eucommia ulmoides* is a traditional Chinese medicinal plant rich in bioactive compounds, particularly chlorogenic acid (CGA). CGA exhibits antioxidant, antiapoptotic, and anti-inflammatory properties and has been shown to improve intestinal health and growth performance in weaned piglets [27,28]. The CGA-rich extract from *Eucommia ulmoides* (CAE) therefore represents a promising antioxidant feed additive. Recent research has indicated that dietary supplementation with CAE before embryo implantation enhances sow reproductive performance primarily through improved antioxidant capacity, immune function and gut microbiota modulation [29]. Notably, CGA exhibits prebiotic-like effects, selectively promoting the growth of beneficial gut microbiota while inhibiting pathogenic species [30]. However, whether maternal CAE supplementation during late gestation and lactation affects offspring gut microbiota colonization and subsequent growth performance through the proposed maternal–milk–piglet axis remains to be determined.

Building upon this evidence, this study employed a randomized controlled feeding design to investigate the effects of dietary CAE supplementation during late gestation and lactation on maternal and offspring outcomes. Measured outcomes encompassed maternal health (oxidative status, immune markers), placental morphology, colostrum composition, offspring birth weight, postnatal growth, and intestinal morphology. Additionally, untargeted metabolomic and 16S rRNA sequencing analyses were performed to characterize colostrum metabolic profiles and fecal microbiota structure, respectively. This design permits causal attribution of treatment effects while identifying potential mechanisms requiring further validation.

## 2. Materials and Methods

### 2.1. Experimental Animals and Study Design

Sixty late-gestating (day 84 of gestation) Dongliao black sows with similar body weights, ages (2–3 years), parities (2–3), and previous reproductive performance were selected and randomly allocated to 2 groups (*n* = 30 per group), namely, the control group (CON) and the CAE group. To minimize the potential confounding effect of maternal age on placental oxidative status, all sows were selected within a narrow and consistent age range. Sows were housed in individual gestation stalls within six blocks per treatment (5 sows/block) for spatial organization; however, each sow was fed individually, with CAE-supplemented or control feed delivered directly to individual feed troughs. Parities and block locations were balanced between the two treatment groups. Sows were housed in these stalls from gestational day 85 until one week before expected farrowing, then moved to individual farrowing crates within the same facility. The experimental period lasted from gestational day 85 to postpartum day 21. Sows in the CON group were fed a basal diet, whereas those in the CAE group received a basal diet supplemented with 2000 mg/kg CAE. This dose was selected based on our previous study [29], which demonstrated that 2000 mg/kg CAE was optimal for improving sow reproductive performance during the peri-implantation period. CAE (STACHA-221015001) was supplied by Changsha Shanghe Biotechnology Co., Ltd. (Changsha, China). High-performance liquid chromatography (HPLC) confirmed that the total CGA content was 51.71%. The term effective CGA content refers to the total quantifiable CGA fraction by HPLC, which serves as the standardization marker for batch-to-batch consistency of the CAE preparation. Heavy metals were below the specifications (Pb ≤ 1.00 ppm, As ≤ 1.00 ppm; total heavy metals ≤ 10.00 ppm). The microbial safety indicators were below the thresholds (total number of aerobic bacteria ≤ 3000 cfu/g, yeast and mold ≤ 300 cfu/g), and *Salmonella* and *Escherichia coli* were not detected. The basal diet composition and nutrient levels are shown in Table 1. During the experimental period, the sows were fed at 06:30 and 17:30 daily and were given free access to water. The feed allowances were as follows: 2.8–3.5 kg/day from gestational days 85–112, 1.5–2.0 kg/day from day 112 to farrowing, and 5.5–6.5 kg/day during lactation. Piglets were grouped according to their respective sow treatments. Immunization, deworming procedures, and other management practices for both the sows and piglets were conducted in accordance with the standard management protocols of the experimental facility. Sows were blocked by parity and randomly allocated to treatments. After completion of the 24 h colostrum intake estimation period, litters were standardized to 9–11 piglets by cross-fostering within the same treatment group to avoid confounding. The Piglet sex ratio was balanced across groups. No creep feed was provided during the lactation period. Birth order was recorded but not included as a covariate because litter standardization and within-treatment cross-fostering effectively distributed these effects evenly across groups, minimizing systematic bias.

### 2.2. Sample Collection

At parturition, 3 sows per block were randomly selected for placental collection (*n* = 18 per group, 3 sows/block × 6 blocks). One placenta was randomly collected from each selected sow. For histological examination, full-thickness tissue samples were excised from the central region of each placenta (avoiding the marginal zone), washed with PBS, and fixed in 4% paraformaldehyde. Blood samples were then collected from the same sows via anterior vena cava puncture using a sterile disposable needle. To minimize stress, sows were gently restrained without chemical sedation; this approach was chosen to avoid potential drug residues in milk and metabolic interference with oxidative stress biomarkers. The puncture site was disinfected with alcohol before and after collection, and sampling was completed as quickly as possible to reduce handling stress. Samples were centrifuged at 4500× *g* for 10 min at 4 °C to obtain serum, which was aliquoted and stored at −80 °C. From these 18 sows, 2 per block were randomly selected for additional colostrum collection (*n* = 12 per group). Colostrum was collected within 6 h postpartum to capture peak IgG concentration; the first three pairs of teats were disinfected with alcohol, and colostrum was collected into sterile centrifuge tubes, aliquoted, and stored at −80 °C. Individual piglet colostrum intake during the first 24 h postpartum was estimated using the predictive equation of Theil et al. [31], incorporating birth weight, 24 h weight gain, and suckling duration; litter-mean colostrum intake was subsequently calculated for statistical analysis (*n* = 12 per group).

For piglet sampling, one piglet per litter was randomly selected from all the 18 L for blood collection at weaning (21 days of age) via anterior vena cava puncture and processed as described for the sows (*n* = 18 per group). From the 12 L in which colostrum was collected, one piglet per litter was randomly selected for fecal collection at weaning, snap-frozen in liquid nitrogen, and stored at −80 °C (*n* = 12 per group). Furthermore, 10 piglets per group (1 piglet per sow, randomly selected from these 12 L of colostrum-collected sows) were selected by a technician blinded to treatment allocation, stratified by body weight to ensure representation across the weight distribution, and humanely euthanized by intravenous injection of sodium pentobarbital (100 mg/kg) into the cranial vena cava following deep anesthesia induced by intramuscular ketamine (10 mg/kg) and xylazine (2 mg/kg). Anesthetic depth was confirmed by loss of righting reflex and unresponsiveness to toe-pinch prior to pentobarbital administration, and euthanasia was verified by cessation of heartbeat. The heart, liver, spleen, lungs, and kidneys were excised and weighed to calculate organ coefficients using the following formula: organ coefficient (%) = (fresh organ weight/live body weight at necropsy) × 100. Duodenal tissue samples were collected from a consistent segment approximately 5–8 cm distal to the pylorus (proximal to the major duodenal papilla) and fixed in 4% paraformaldehyde for histomorphological analysis.

### 2.3. Assessment of Offspring Birth Weight and Piglet Growth Performance

Offspring birth weight was evaluated in 30 sows per group using litter-mean birth weight (kg), calculated as the total birth weight of all piglets born alive divided by the number of piglets born alive per litter. Piglet growth performance was assessed by recording individual body weights at birth and at weaning. The average daily gain (ADG, kg/d) from birth to weaning was calculated for each piglet as (body weight at weaning − body weight at birth)/21 and subsequently averaged at the litter level for statistical analysis (*n* = 30 L per group).

### 2.4. Biochemical Analyses

Approximately 0.1 g of placental tissue was homogenized in 1 mL of ice-cold extraction buffer at 4 °C, followed by centrifugation at 12,000× *g* for 10 min at 4 °C. The resulting supernatants were collected for analysis. Superoxide dismutase (SOD), catalase (CAT), and glutathione peroxidase (GPx) activities in placental tissue were measured using commercial assay kits purchased from Nanjing Addison Biotechnology Co., Ltd. (Nanjing, China). Serum SOD and CAT activities in sows and piglets were determined using the same kits. Serum total antioxidant capacity (T-AOC) and malondialdehyde (MDA) levels were analyzed using assay kits from Beijing Box Biotechnology Co., Ltd. (Beijing, China). For immunoglobulin analysis, IgA, IgG, and IgM were measured in sow and piglet sera, and IgG was measured in colostrum, using kits from Fankewei Biotechnology Co., Ltd. (Wuhan, China).

### 2.5. Histomorphological Analysis of the Sow Placenta and Piglet Duodenum

The tissue samples were fixed in 4% paraformaldehyde solution for 24–48 h, washed under running water for 2 h, dehydrated through graded ethanol (75% for 4 h, 85% for 2 h, 90% for 1.5 h, 95% for 1 h, and absolute ethanol for 1 h), cleared in xylene twice (30 min each), and embedded in paraffin. Sections were cut at a thickness of 4 μm, floated in a 42 °C water bath, and heated at 60 °C for 3 h. Following conventional deparaffinization and rehydration, the sections were subjected to hematoxylin and eosin (H&E) staining as follows: hematoxylin (Sigma–Aldrich, St. Louis, MO, USA) staining for 5 min, differentiation in 1% acid alcohol for 2–5 s, bluing in warm water, eosin (Sinopharm, Beijing, China) staining for 2 min, rapid dehydration through graded ethanol, clearing in xylene, and mounting with neutral balsam (Sinopharm, Beijing, China). Observation and imaging were performed using an Olympus BX53 microscope (Olympus Corporation, Tokyo, Japan); placental tissues were examined at 100× and 400× magnification, while piglet duodenal tissues were examined at 50×. Morphometric analysis was conducted using ImageJ v1.53k software. For each placenta, four structurally intact villus cross-sections were selected by systematic random sampling and viewed at 400× magnification. Capillaries with clear lumens were counted and villus circumference (μm) was measured in each section to calculate villus capillary density (number/mm) as follows: (capillary number × 1000)/villus circumference (μm). The mean of the four measurements was used as the final value for statistical analysis. For the duodenum, six well-oriented, morphologically intact villi per sample were selected. Villus height (VH, μm; vertical distance from the villus tip to the crypt opening) and crypt depth (CD, μm; vertical distance from the crypt opening to the bottom) were measured in each villus, and the villus height to crypt depth ratio (VH/CD) was calculated. The mean of the six measurements was used as the final value for statistical analysis.

### 2.6. 16S rRNA Sequencing of the Fecal Microbiota

Each fecal sample was transferred to a centrifuge tube containing lysis buffer for grinding and homogenization. Total microbial DNA was extracted using an OMEGA Soil DNA Kit (OMEGA Biotek, Norcross, GA, USA), and the sample was mixed with 500 mg of glass beads and 0.8 mL of Buffer SLX Mlus and vortexed for 1–5 min. Then, 80 μL of Buffer DS was added, and the sample was incubated at 70 °C for 10 min (with twice inversions during incubation) and centrifuged at 13,000× *g* for 5 min, after which 600 μL of the supernatant was collected. Next, 200 μL of Buffer SP2 and 100 μL of HTR Reagent were sequentially added, after which the sample was incubated on ice for 5 min before centrifugation. A 400 μL aliquot of the supernatant was transferred and subjected to magnetic bead adsorption, after which the sample was washed, dried, and eluted with 50 μL of elution buffer (incubation at 65 °C for 15 min). DNA integrity and purity were assessed by 0.8% agarose gel electrophoresis and a Nanodrop spectrophotometer, respectively.

The V3-V4 hypervariable regions of the bacterial 16S rRNA gene were amplified using the primer pair 338F (5′-ACTCCTACGGGAGGCAGCA-3′) and 806R (5′-GGACTACHVGGGTWTCTAAT-3′). PCR was performed under the following thermal cycling conditions: initial denaturation at 98 °C for 5 min; followed by 25 cycles of 98 °C for 30 s (denaturation), 52 °C for 30 s (annealing), and 72 °C for 45 s (extension); and a final extension at 72 °C for 5 min. Amplicons were visualized on a 2% agarose gel, purified using AMPure XP beads (Beckman Coulter, Brea, CA, USA), and subsequently used for library construction. The sequencing libraries were subjected to quality control on an Agilent 2100 bioanalyzer (Agilent Technologies, Santa Clara, CA, USA) and then sequenced on an Illumina NovaSeq platform (Illumina, San Diego, CA, USA) with 2 × 250 bp paired-end reads at a loading concentration of 15–18 pM. The raw sequencing run yielded approximately 3.2 million paired-end reads across all 24 samples (mean: 133,454 ± 6441 reads per sample; range: 109,804–141,217). After demultiplexing and adapter trimming using QIIME 2 q2-demux (version 2024.5) and cutadapt (version 2.3), respectively, an average of 125,015 ± 6283 reads per sample remained. DADA2 (version 1.32) was used for quality filtering, denoising, merging, and chimera removal to generate amplicon sequence variants (ASVs) and abundance tables. Quality filtering was performed with parameters optimized by the sequencing service provider (Personalbio, Shanghai, China): forward and reverse reads were truncated at positions where the Phred quality score dropped below 20, and reads with >2 expected errors were discarded. Paired-end reads were merged with a minimum overlap of 12 bp, and chimeric sequences were removed using the consensus method. An average of 82,212 ± 8490 high-quality reads per sample were retained (retention rate: 61.6%; range: 65,608–100,492). Good’s coverage exceeded 0.97 across all samples, indicating adequate sequencing depth. Negative extraction controls (blank DNA extractions, *n* = 2) and PCR negative controls (no-template reactions, *n* = 2) were included in each batch; contaminant ASVs were inspected and excluded from downstream analysis. Taxonomic annotation was performed on the basis of the Greengenes2 database [32]. Alpha diversity was assessed at the ASV level using Chao1 (richness), Shannon (diversity), Simpson diversity (1-D; higher values indicate greater diversity, with greater sensitivity to dominance by individual ASVs) and Pielou_e (evenness). Between-group comparisons of alpha diversity indices were performed using Student’s *t*-test or Mann–Whitney U test. Benjamini–Hochberg FDR correction was applied across the four indices to control Type I error inflation, with significance declared at FDR *q* < 0.05. Beta diversity was assessed using Bray–Curtis dissimilarity matrices calculated from the ASV relative abundance data and visualized by principal coordinate analysis (PCoA) and hierarchical clustering. Group differences were assessed using analysis of similarity (ANOSIM) with 999 permutations. An R > 0 indicates greater between-group than within-group variation, and statistical significance was determined at *p* < 0.05. Linear discriminant analysis effect size (LEfSe) was employed to identify taxa exhibiting differential abundance at both the phylum and genus levels. The data were normalized to 1,000,000 reads per sample, and taxa whose relative abundance was <0.05% were excluded. Differential abundance between the two groups was initially assessed using the Wilcoxon rank-sum test, followed by LDA effect size estimation. Biomarkers were retained if they met both LDA > 4.0 and FDR *q* < 0.05.

### 2.7. Untargeted Metabolomic Analysis of Colostrum

An aliquot of 100 μL of sow colostrum was transferred to a 2 mL centrifuge tube, followed by the addition of 400 μL of a precooled methanol–acetonitrile mixture (1:1, *v*/*v*, LC–MS grade). The mixture was vortexed for 30 s, incubated at −20 °C for 30 min, and then centrifuged at 12,000 rpm for 10 min at 4 °C. The supernatant was collected and dried under vacuum to near dryness and then reconstituted in 150 μL of 50% aqueous methanol containing 5 ppm 2-chloro-L-phenylalanine (Sigma–Aldrich, St. Louis, MO, USA) as an internal standard. After vortexing for 30 s and centrifugation, the supernatant was filtered through a 0.22 μm membrane filter and collected in an autosampler vial. For quality control (QC), 10–20 μL aliquots from each sample filtrate were pooled in equal volumes. Chromatographic separation was performed on a Waters ACQUITY UPLC H-Class system (Waters Corporation, Milford, MA, USA) equipped with an ACQUITY UPLC HSS T3 column (100 Å, 1.8 µm, 2.1 mm × 100 mm) using 0.1% formic acid in water and 0.1% formic acid in acetonitrile as the mobile phases for gradient elution. The flow rate was 0.4 mL/min, the column temperature was 40 °C, the autosampler temperature was 8 °C, and the injection volume was 2 μL. Mass spectrometry detection was conducted using a Thermo Orbitrap Exploris 120 mass spectrometer (Thermo Fisher Scientific, San Jose, CA, USA) under the control of Xcalibur software (version 4.7) for data-dependent acquisition (DDA) in both positive and negative ion modes. The heated electrospray ionization (HESI) source parameters were as follows: spray voltage of +3.5 kV/−3.0 kV, sheath gas flow rate of 40 arbitrary units (arb), auxiliary gas flow rate of 15 arb, capillary temperature of 325 °C, and auxiliary gas heater temperature of 300 °C. For the MS1 full scan, the resolution was 60,000, with a scan range of *m*/*z* 100–1000. For MS/MS, the 4 most intense ions were selected for higher-energy collisional dissociation (HCD) fragmentation with a dynamic exclusion time of 8 s, a resolution of 15,000, and stepped collision energies of 20%, 40%, and 60%.

The raw data were imported into Compound Discoverer 3.3 (Thermo Fisher Scientific, Waltham, MA, USA) for peak extraction, alignment, retention time correction, and total ion current (TIC) normalization. For quality control (QC), 10–20 μL aliquots from each sample filtrate were pooled in equal volumes. QC samples were injected every 10 samples throughout the LC–MS run to monitor analytical stability. Pearson correlation coefficients among QC injections exceeded 0.95, indicating high analytical consistency. Across QC injections, 70% of metabolite features exhibited relative standard deviation (RSD) < 15% and 85% exhibited RSD < 20%, demonstrating satisfactory analytical reproducibility. Features with missing values in >50% of QC samples or RSD > 30% were excluded; remaining missing values across all samples were imputed using the Fill-Gaps algorithm with a minimum of 2 adjacent scans. Metabolite annotation was performed by searching exact masses (±15 ppm) and MS/MS spectra against the Human Metabolome Database (HMDB; version 5.0) using Compound Discoverer 3.3. The software generates a composite Total score (0–100) integrating MS1 mass accuracy, isotope pattern matching, and MS/MS spectral similarity. Annotations were tiered by confidence based on Total score and manual MS/MS inspection; only high- and medium-confidence annotations were retained for statistical analysis. For univariate analysis, metabolite peak intensities were log_2_-transformed. Each metabolite was tested using linear mixed models (LMM) with treatment and parity as fixed effects and block as a random effect, consistent with the statistical approach for other sow-level outcomes (see Section 2.8). *p*-values were corrected using the Benjamini–Hochberg FDR method. Differentially abundant metabolites were defined as *q* < 0.05 and |log_2_FC| > 1.0. Effect sizes are reported as fold changes with 95% confidence intervals. Positive and negative ion mode data were processed separately; for metabolites detected in both modes, the adduct with higher intensity and annotation confidence was retained, and the final dataset was consolidated to unique metabolite identifiers to avoid redundancy. Pathway enrichment analysis of these differentially abundant metabolites was performed using the Kyoto Encyclopedia of Genes and Genomes (KEGG) database.

### 2.8. Statistical Analysis

Statistical analyses were performed using IBM SPSS Statistics (version 26.0), GraphPad Prism (version 8.0), and R software (version 4.3.3). Data are presented as means ± standard error of the mean (SEM). ADG was designated a priori as the single primary endpoint and analyzed separately at *p* < 0.05 without multiple comparison correction. Sow-level outcomes (including ADG) were analyzed using LMM with treatment and parity as fixed effects and block as a random effect. The treatment × parity interaction was non-significant (*p* > 0.10) and removed from the final model. Benjamini–Hochberg FDR correction was applied across all sow-level parameters except ADG within each biological family; significance was set at FDR *q* < 0.05. For piglet-level outcomes, one piglet per litter was randomly selected, and independent two-group comparisons were performed using Student’s *t* test, Welch’s *t* test, or the Mann–Whitney U test, as appropriate, following assessment of normality (Shapiro–Wilk test) and variance homogeneity (Levene’s test). Benjamini–Hochberg FDR correction was applied within each parameter family; significance was declared at FDR *q* < 0.05. Partial Spearman rank correlation controlling for treatment was performed to examine relationships among maternal antioxidant parameters and litter-mean birth weight, colostrum composition and piglet growth performance, and colostrum composition and VH/CD. Benjamini–Hochberg FDR correction was applied across all correlation coefficients within each relationship family; significance was set at FDR *q* < 0.05. Detailed analytical parameters for 16S rRNA sequencing and untargeted metabolomics are provided in the respective sections above.

## 3. Results

### 3.1. Effects of Dietary CAE on Offspring Birth Weight, Colostrum Parameters, and Piglet Growth Performance

**Figure 1:**
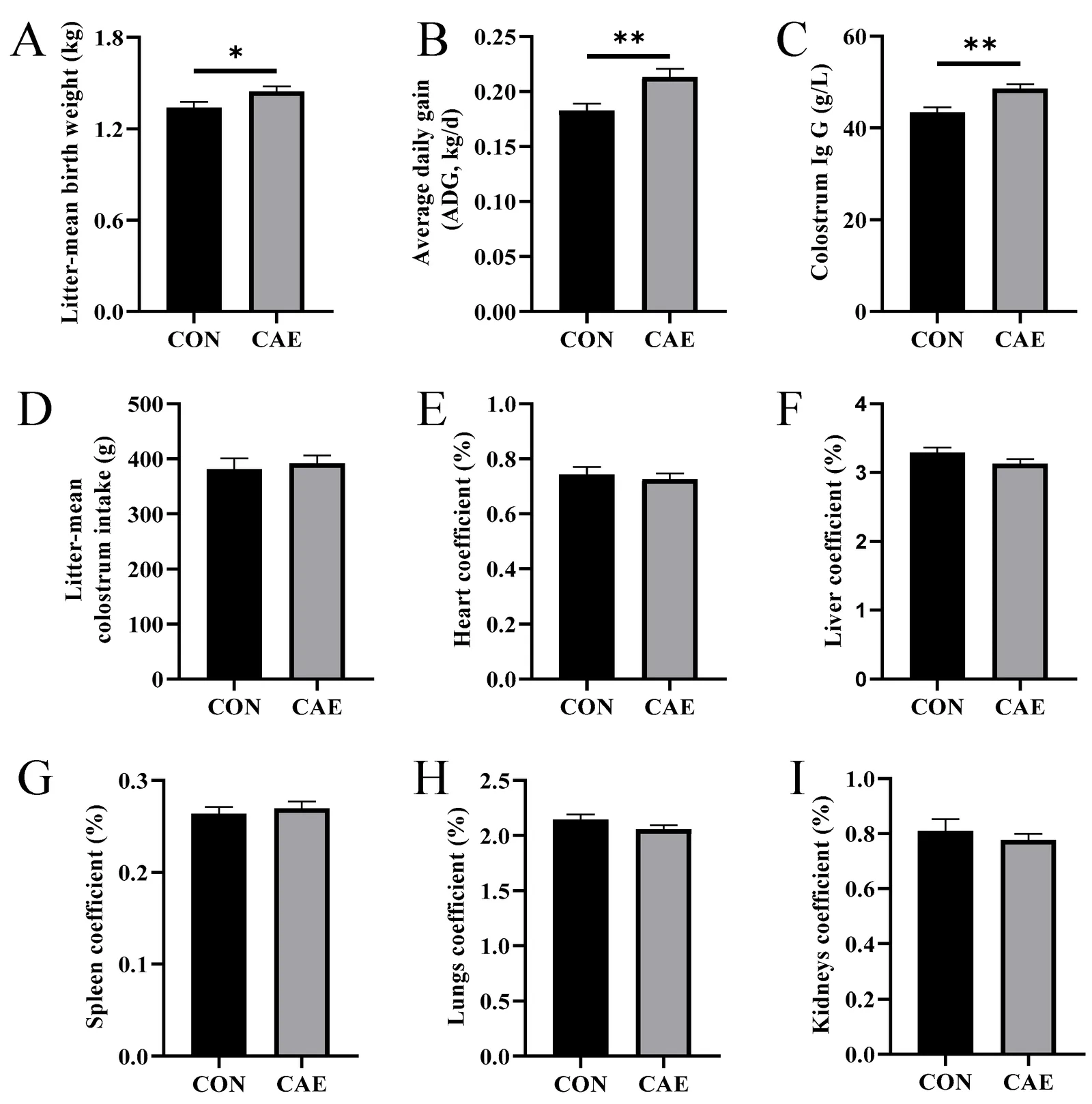
Effects of dietary supplementation with CAE on offspring birth weight, colostrum parameters, and piglet growth performance. Litter-mean birth weight (*n* = 30 sows per group) (**A**). ADG from birth to weaning (*n* = 30 L per group) (**B**). Colostrum parameters (*n* = 12 sows per group): colostrum IgG (**C**) and litter-mean colostrum intake (**D**). Organ coefficients (*n* = 10 piglets per group) of the heart (**E**), liver (**F**), spleen (**G**), lungs (**H**), and kidneys (**I**). Organ coefficient (%) = (fresh organ weight/live body weight at necropsy) × 100. CON, sows fed a basal diet; CAE, sows fed a basal diet supplemented with 2000 mg/kg CAE. The data are presented as the mean ± SEM. ** *p* < 0.01 for ADG; * *q* < 0.05, ** *q* < 0.01 for all other parameters.

Compared with the CON group, the CAE supplementation significantly increased the litter-mean birth weight (*q* < 0.05) (Figure 1A). During lactation, the ADG of the piglets in the CAE group was significantly greater than that in the CON group (*p* < 0.01) (Figure 1B). CAE supplementation was also associated with increased colostrum IgG concentration (*q* < 0.01) (Figure 1C). However, litter-mean colostrum intake did not differ significantly between groups (*q* > 0.05) (Figure 1D). Furthermore, no significant differences in the heart, liver, spleen, lung, or kidney coefficients were detected between the two groups (*q* > 0.05) (Figure 1E–I).

### 3.2. Effects of Dietary CAE on Piglet Intestinal Morphology

**Figure 2:**
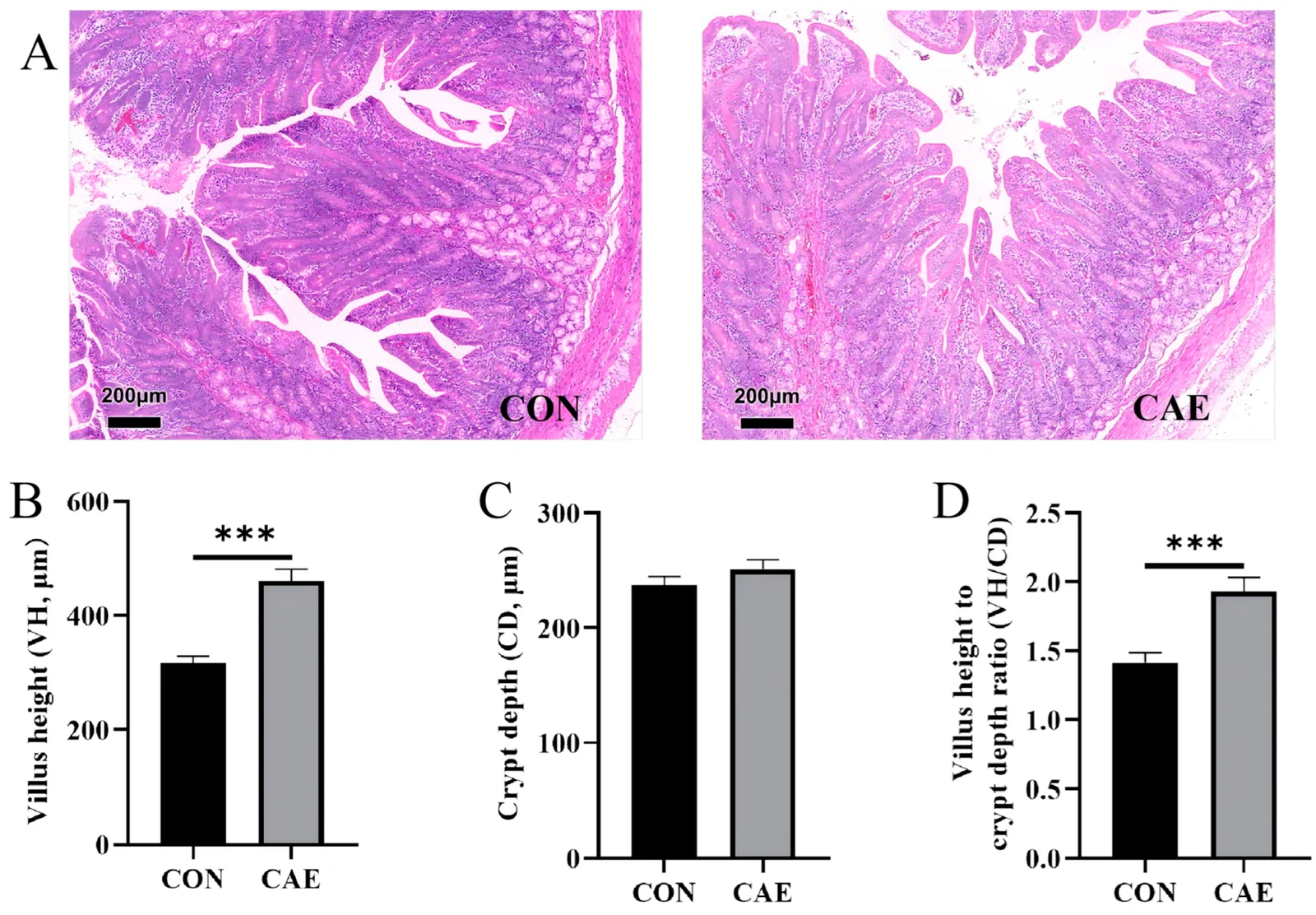
Effects of dietary supplementation with CAE on piglet duodenal morphology. Representative H&E-stained sections of the duodenum (**A**), quantitative analysis of the VH (**B**), CD (**C**), and VH/CD (**D**). CON, sows fed a basal diet; CAE, sows fed a basal diet supplemented with 2000 mg/kg CAE. The data are presented as the mean ± SEM (*n* = 10 piglets per group). *** *q* < 0.001.

In addition to growth performance parameters, intestinal morphology during development was assessed by H&E staining to evaluate the effects of maternal CAE supplementation on offspring gut health. As shown in Figure 2A–D, compared with the CON group, the CAE group exhibited significantly greater duodenal VH (*q* < 0.001) and VH/CD (*q* < 0.001), whereas the CD did not differ significantly (*q* > 0.05).

### 3.3. Effects of Dietary CAE on Serum Antioxidant and Immune Parameters in Sows

**Figure 3:**
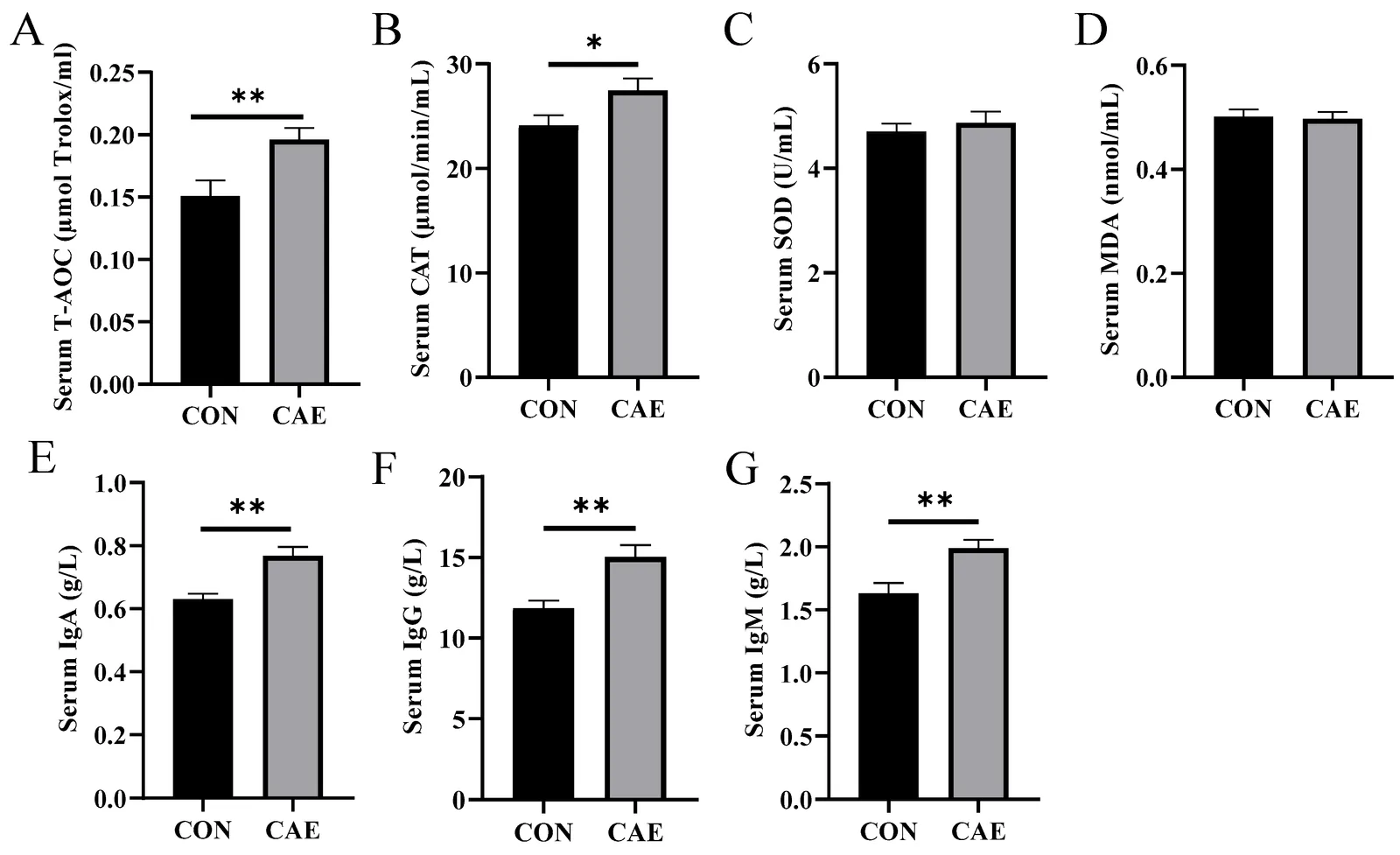
Effects of dietary CAE supplementation on serum antioxidant status and immune function in sows at parturition (day 0 postpartum). Serum T-AOC (**A**), CAT (**B**) and SOD (**C**) activities, MDA (**D**) content, and IgA (**E**), IgG (**F**), and IgM (**G**) concentrations in sows. CON, sows fed a basal diet; CAE, sows fed a basal diet supplemented with 2000 mg/kg CAE. The data are presented as the mean ± SEM (*n* = 18 sows per group). * *q* < 0.05, ** *q* < 0.01.

Compared with the CON treatment group, the CAE group presented significantly higher sow serum T-AOC (*q* < 0.01) and CAT (*q* < 0.05) activities; however, the SOD activity and MDA content did not significantly differ between the groups (*q* > 0.05) (Figure 3A–D). Additionally, serum IgA (*q* < 0.01), IgG (*q* < 0.01), and IgM (*q* < 0.01) levels were significantly higher in the CAE group (Figure 3E–G).

### 3.4. Effects of Dietary CAE on Piglet Serum Antioxidant and Immune Parameters

**Figure 4:**
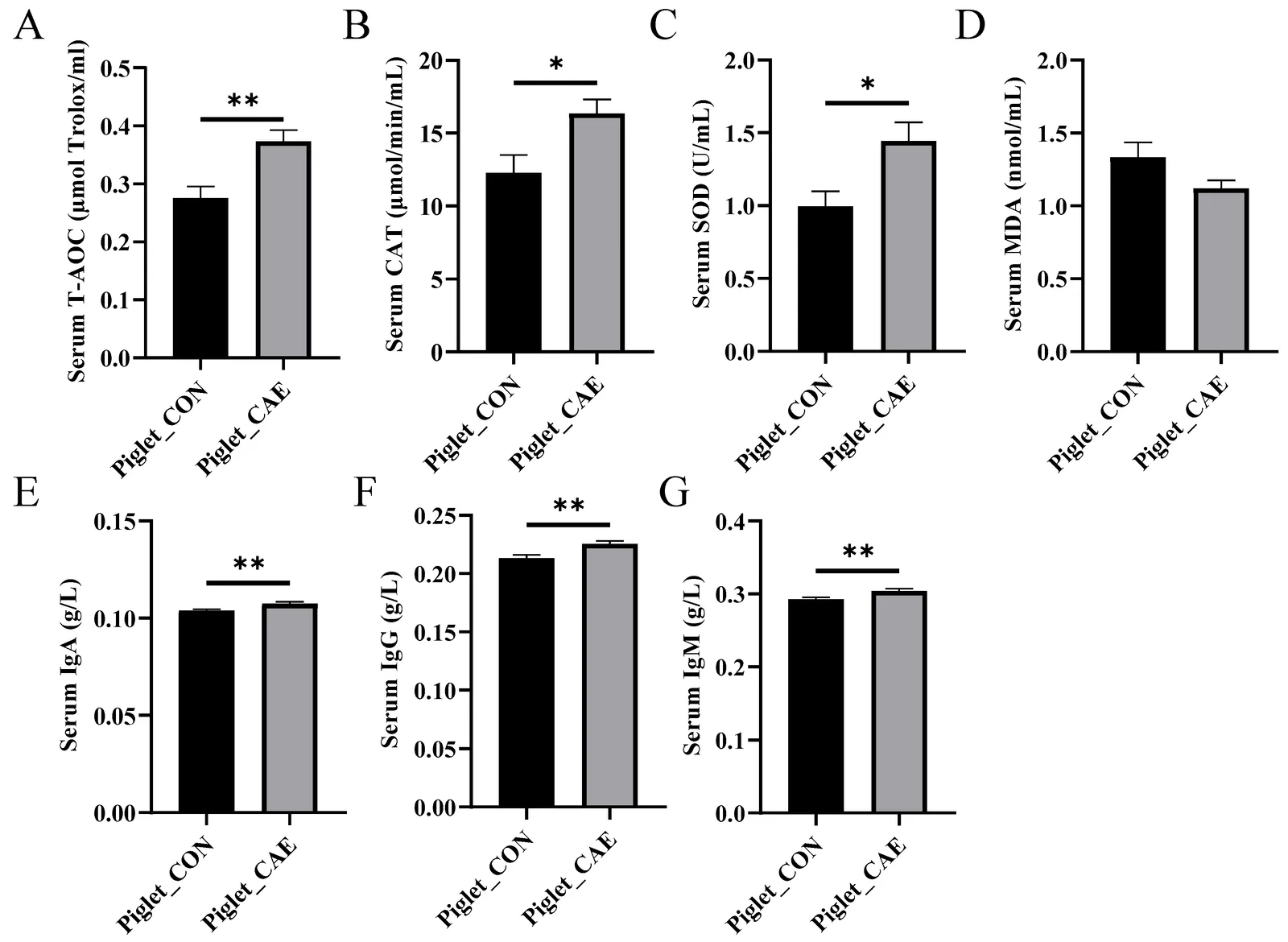
Effects of dietary supplementation with CAE on piglet serum antioxidant and immune parameters at weaning (21 days of age). Serum T-AOC (**A**), CAT (**B**) and SOD (**C**) activities, MDA (**D**) content, and IgA (**E**), IgG (**F**), and IgM (**G**) concentrations in piglets. CON, sows fed a basal diet; CAE, sows fed a basal diet supplemented with 2000 mg/kg CAE. The data are presented as the mean ± SEM (*n* = 18 piglets per group). * *q* < 0.05, ** *q* < 0.01.

To investigate the effects of dietary CAE supplementation in sows on the antioxidant activity and immune performance of their offspring, piglet serum parameters were measured. Consistent with the trends observed in sows, piglets in the CAE group exhibited significantly higher T-AOC (*q* < 0.01), CAT (*q* < 0.05) and SOD (*q* < 0.05) activities, whereas the MDA level did not significantly differ (*q* > 0.05) (Figure 4A–D). Furthermore, serum IgA (*q* < 0.01), IgG (*q* < 0.01), and IgM (*q* < 0.01) levels were significantly greater in the piglets in the CAE group (Figure 4E–G).

### 3.5. Effects of Dietary CAE on Sow Placental Villus Morphology and Antioxidant Parameters

**Figure 5:**
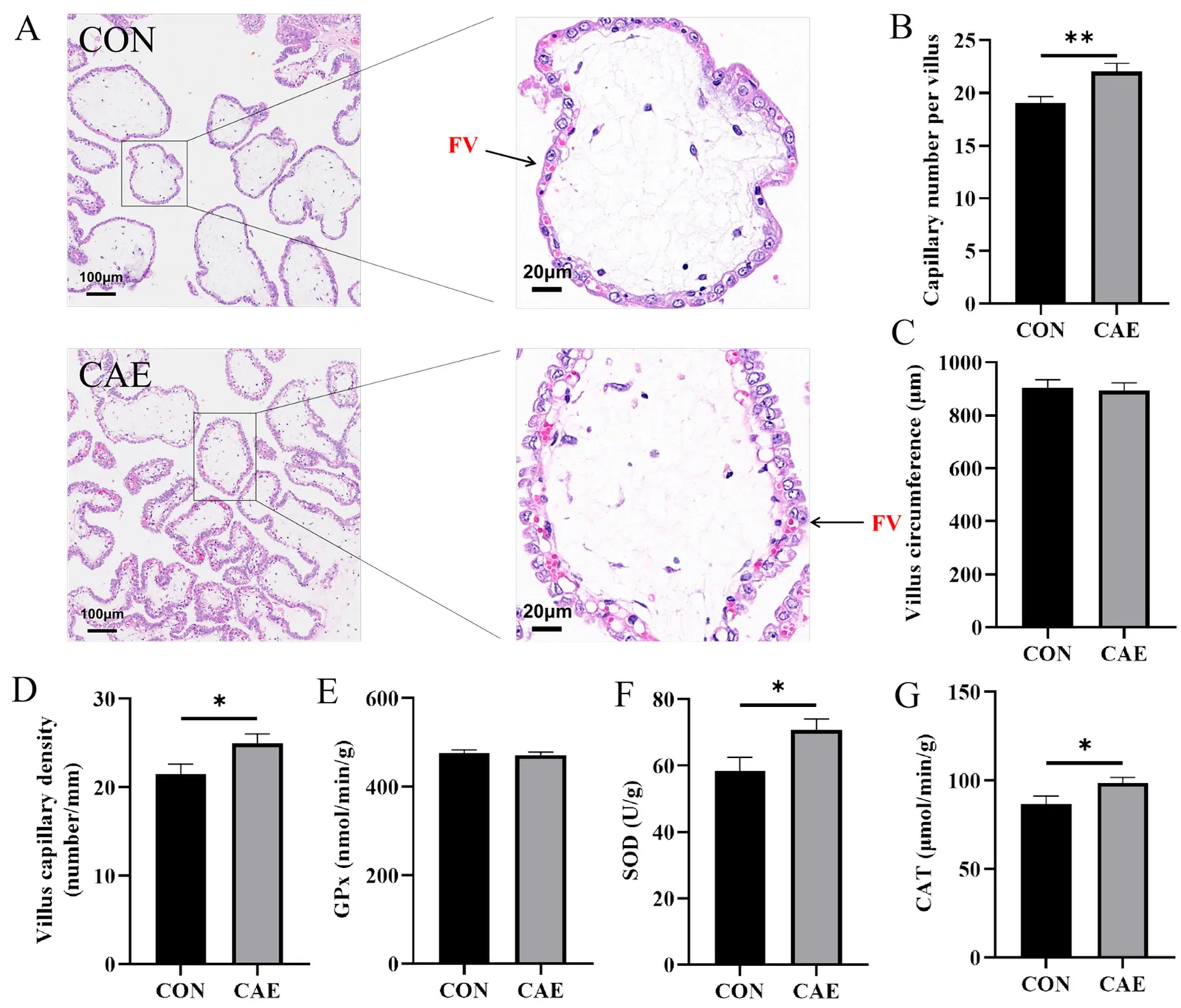
Effects of dietary supplementation with CAE on placental villus morphology and antioxidant capacity. (**A**) Representative H&E-stained sections showing placental villus architecture. FV denotes fetal villous capillaries. Scale bars: 100 μm (**left panels**) and 20 μm (**right panels**); (**B**) capillary number per villus; (**C**) villus circumference (μm); (**D**) villus capillary density (number/mm), calculated as (capillary number × 1000)/villus circumference (μm). Antioxidant enzyme activities in placental tissue (*n* = 18 placentas per group): GPx (**E**), SOD (**F**), and CAT (**G**). CON, sows fed a basal diet; CAE, sows fed a basal diet supplemented with 2000 mg/kg CAE. The data are presented as the mean ± SEM. * *q* < 0.05, ** *q* < 0.01.

Placental vascular morphology was assessed by H&E staining. As shown in Figure 5A–D, compared with the CON group, the CAE group exhibited significant increases in capillary number (*q* < 0.01) and villus capillary density (*q* < 0.01), whereas the difference in the villus circumference was not significant (*q* > 0.05). Placental oxidative status was also evaluated (Figure 5E–G). Compared with the CON group, the CAE group exhibited significantly higher SOD (*q* < 0.05) and CAT (*q* < 0.05) activities in placental tissue, whereas GPx activity did not significantly differ (*q* > 0.05).

### 3.6. Effects of CAE on Piglet Fecal Microbiota Diversity and Composition

**Figure 6:**
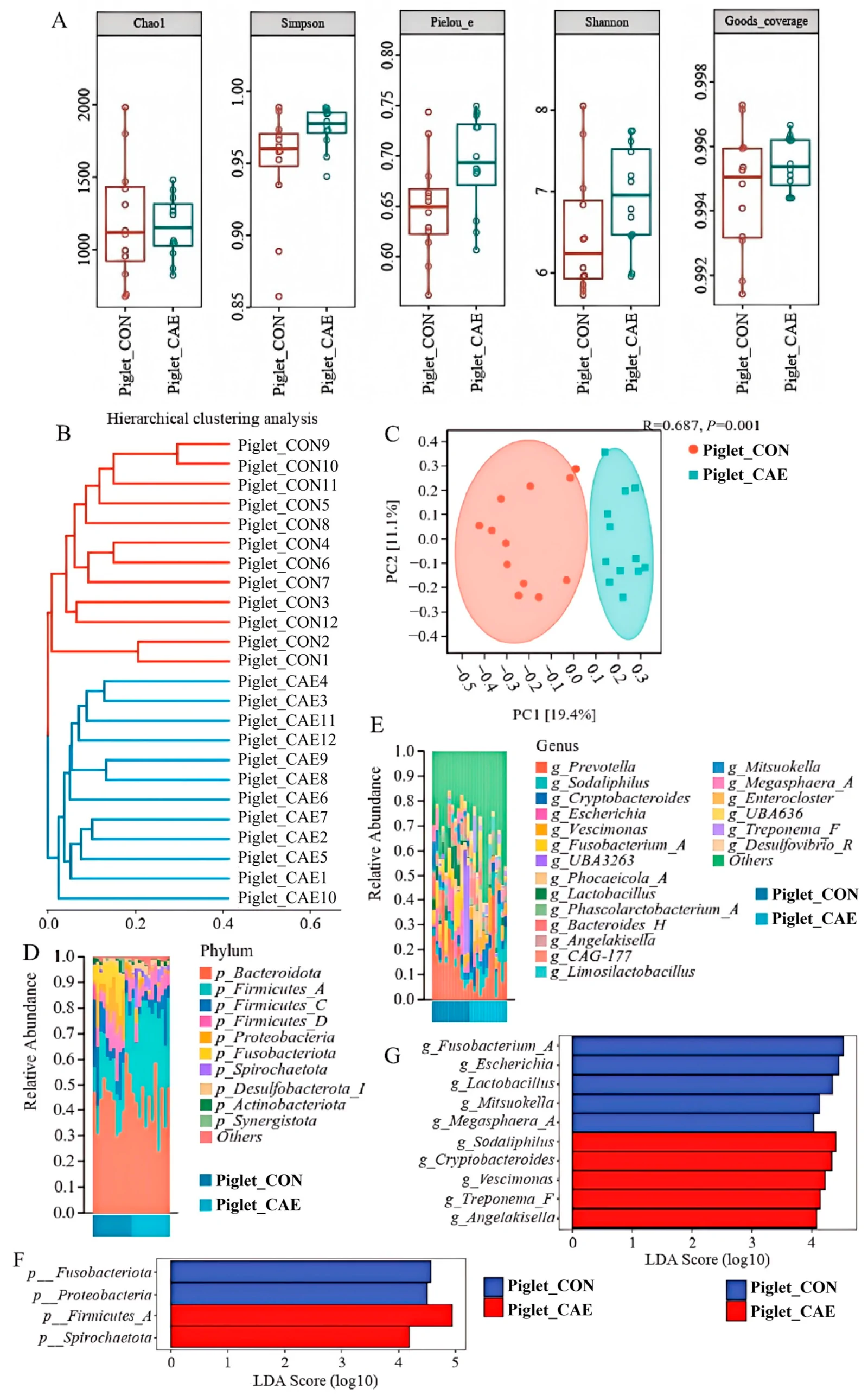
Effects of maternal dietary supplementation with CAE on piglet fecal microbiota diversity and composition (*n* = 12 piglets per group). Alpha diversity indices (Chao1, Simpson, Pielou_e, and Shannon) and sequencing depth coverage (Good’s coverage) (**A**). Hierarchical clustering analysis (**B**). PCoA and ANOSIM (**C**). Relative abundances of the top 10 phyla (**D**) and the top 20 genera (**E**). LEfSe analysis of differentially abundant taxa at the phylum (**F**) and genus (**G**) levels. CON, piglets from sows fed a basal diet; CAE, piglets from sows fed a basal diet supplemented with 2000 mg/kg CAE.

Alpha diversity analysis revealed no significant differences between the CON and CAE groups in any of the evaluated indices, including species richness (Chao1), Shannon diversity, Simpson diversity, and species evenness (Pielou_e index) (*q* > 0.05). Good’s coverage exceeded 0.99 across all samples, confirming adequate sequencing depth (Figure 6A). Beta diversity analysis revealed significant differences between the two groups in the PCoA plot (R = 0.687, *p* = 0.001), with cluster analysis showing distinct clustering patterns (Figure 6B–C). The relative abundance of *Firmicutes_A* was higher in the CAE group (36.1% vs. 16.7%), whereas those of *Firmicutes_D*, *Fusobacteriota*, and *Proteobacteria* were relatively lower (Figure 6D). At the genus level, the CON group was dominated by *Prevotella* (18.5%), *Fusobacterium_A* (7.3%), and *Escherichia* (6.9%), whereas *Prevotella* (11.4%), *Sodaliphilus* (8.7%), *Cryptobacteroides* (6.3%), and *Vescimonas* (5.6%) were predominant in the CAE group (Figure 6E).

LEfSe analysis further identified 14 differentially abundant taxa as candidate biomarkers. *Firmicutes_A*, *Spirochaetota*, *Sodaliphilus*, *Cryptobacteroides*, *Vescimonas Treponema_F*, and *Angelakisella* were significantly enriched in the CAE group, whereas *Fusobacteriota*, *Proteobacteria*, *Fusobacterium_A*, *Escherichia, Lactobacillus*, *Mitsuokella*, and *Megasphaera_A* were significantly enriched in the CON group (Figure 6F, G) (LDA > 4.0, *q* < 0.05).

### 3.7. Effects of Dietary CAE on Differentially Abundant Metabolites in Colostrum

**Figure 7:**
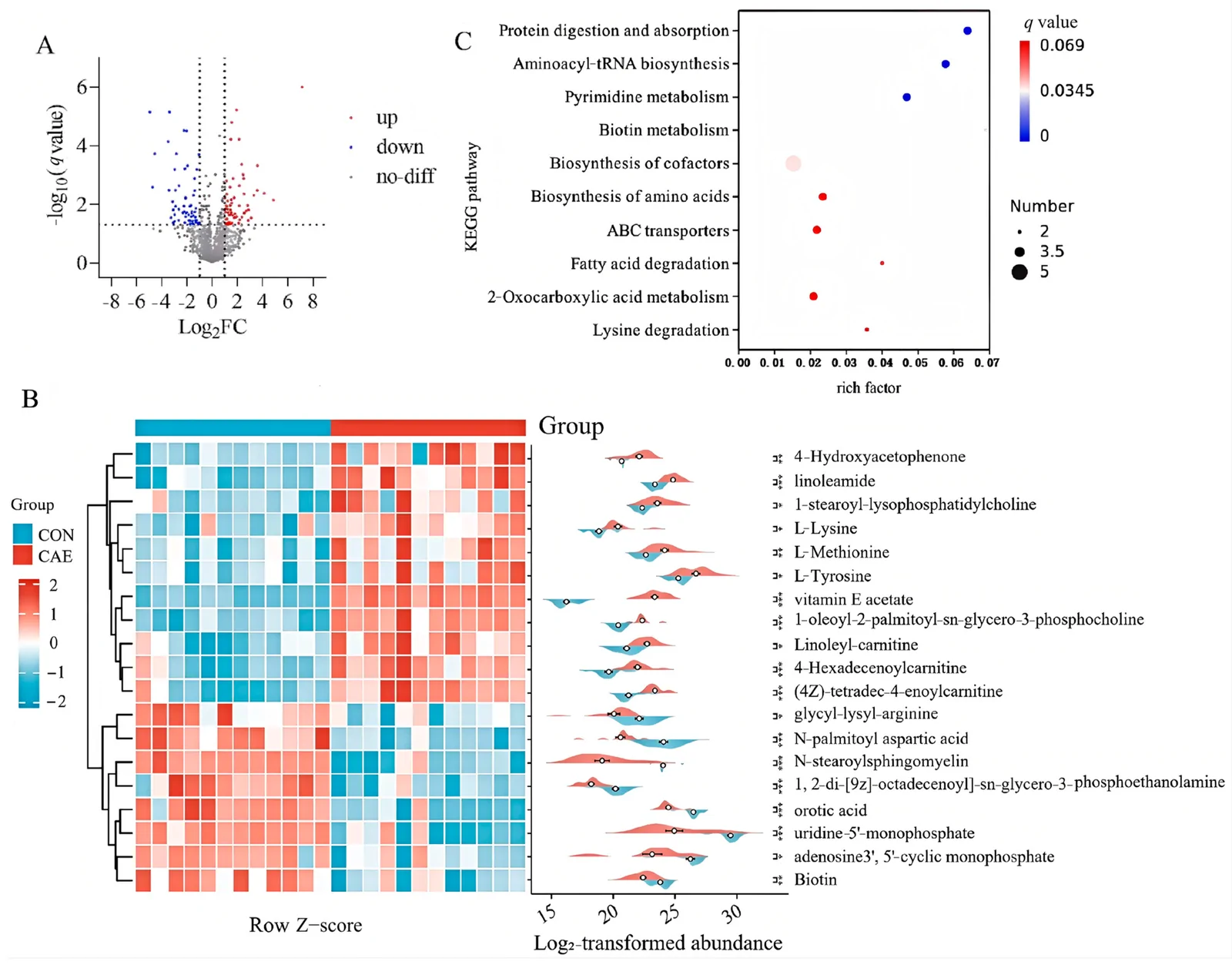
Effects of dietary supplementation with CAE on colostrum metabolites (*n* = 12 sows per group). (**A**) Volcano plot showing the distribution of all detected metabolites; horizontal and vertical dashed lines indicate the significance thresholds, and red and blue dots indicate significantly up-regulated and down-regulated metabolites, respectively (*q* < 0.05 and |log_2_FC| > 1.0). (**B**) Log_2_-transformed abundances of 19 representative differential metabolites, standardized by row Z-score for heatmap visualization, are shown for CON (blue) and CAE (red) groups, with all data log2-transformed prior to statistical analysis; the right panel displays violin plots of the log_2_-transformed abundance distribution for each metabolite; significance levels are indicated as * *q* < 0.05, ** *q* < 0.01, *** *q* < 0.001. (**C**) Bubble chart of the results of the KEGG metabolic pathway enrichment analysis. The *x*-axis indicates the enrichment factor (defined as the ratio of the number of differentially enriched metabolites to the total number of metabolites annotated to that pathway); higher values indicate greater degrees of enrichment. The *y*-axis lists the KEGG pathway names. Bubble size represents the number of differentially abundant metabolites enriched in the corresponding pathway. The color gradient indicates the enrichment significance level (*q* value), where values closer to zero indicate more significant enrichment. CON, sows fed a basal diet; CAE, sows fed a basal diet supplemented with 2000 mg/kg CAE.

A total of 127 differentially abundant metabolites were identified (*q* < 0.05, |log_2_FC| > 1.0), with 66 up-regulated and 61 down-regulated in the CAE group (Figure 7A). Figure 7B presents the heatmap and abundance distributions of 19 representative Tier 1–2 annotated metabolites across major metabolic classes. The significantly increased metabolites included antioxidants and phenolic compounds such as vitamin E acetate and 4-hydroxyacetophenone; acylcarnitines involved in fatty acid oxidation and mitochondrial energy metabolism such as 4-hexadecenoylcarnitine, linoleyl-carnitine, and (4Z)-tetradec-4-enoylcarnitine; fatty acid amides with signaling functions such as linoleamide; and amino acids such as L-lysine, L-methionine, and L-tyrosine. The significantly decreased metabolites included pyrimidine metabolism intermediates such as orotic acid and uridine-5′-monophosphate (UMP); cyclic nucleotide signaling molecules such as adenosine 3′,5′-cyclic monophosphate (cAMP); bioactive peptides such as glycyl-lysyl-arginine; and cofactor-related vitamins such as biotin. Additionally, membrane lipid remodeling was observed. The levels of lysophosphatidylcholine (LPC; 1-stearoyl-lysophosphatidylcholine) and phosphatidylcholine (PC; 1-oleoyl-2-palmitoyl-sn-glycero-3-phosphocholine) significantly increased, whereas the levels of phosphatidylethanolamine (PE; 1,2-di-[(9Z)-octadecenoyl]-sn-glycero-3-phosphoethanolamine) and sphingomyelin (N-stearoylsphingomyelin) decreased. Notably, N-palmitoyl aspartic acid, an N-acyl amino acid with emerging roles in metabolic signaling, was also significantly decreased.

KEGG metabolic pathway enrichment analysis, performed using all differential metabolites with available KEGG IDs, revealed five significantly enriched pathways (*q* < 0.05; Figure 7C). Protein digestion and absorption and aminoacyl-tRNA biosynthesis were predominantly driven by up-regulated amino acids, including L-lysine, L-methionine, and L-tyrosine. Pyrimidine metabolism was exclusively associated with down-regulated metabolites, including UMP and orotic acid. Biotin metabolism and biosynthesis of cofactors exhibited mixed regulatory patterns, with biotin and orotic acid being down-regulated, whereas L-lysine, L-methionine, and L-tyrosine were up-regulated. Five additional pathways showed marginal enrichment (0.05 < *q* < 0.07), including biosynthesis of amino acids, ABC transporters, fatty acid degradation, 2-oxocarboxylic acid metabolism, and lysine degradation.

### 3.8. Partial Correlations of Maternal Antioxidant Status and Colostrum Composition with Reproductive Outcomes and Offspring Performance

**Figure 8:**
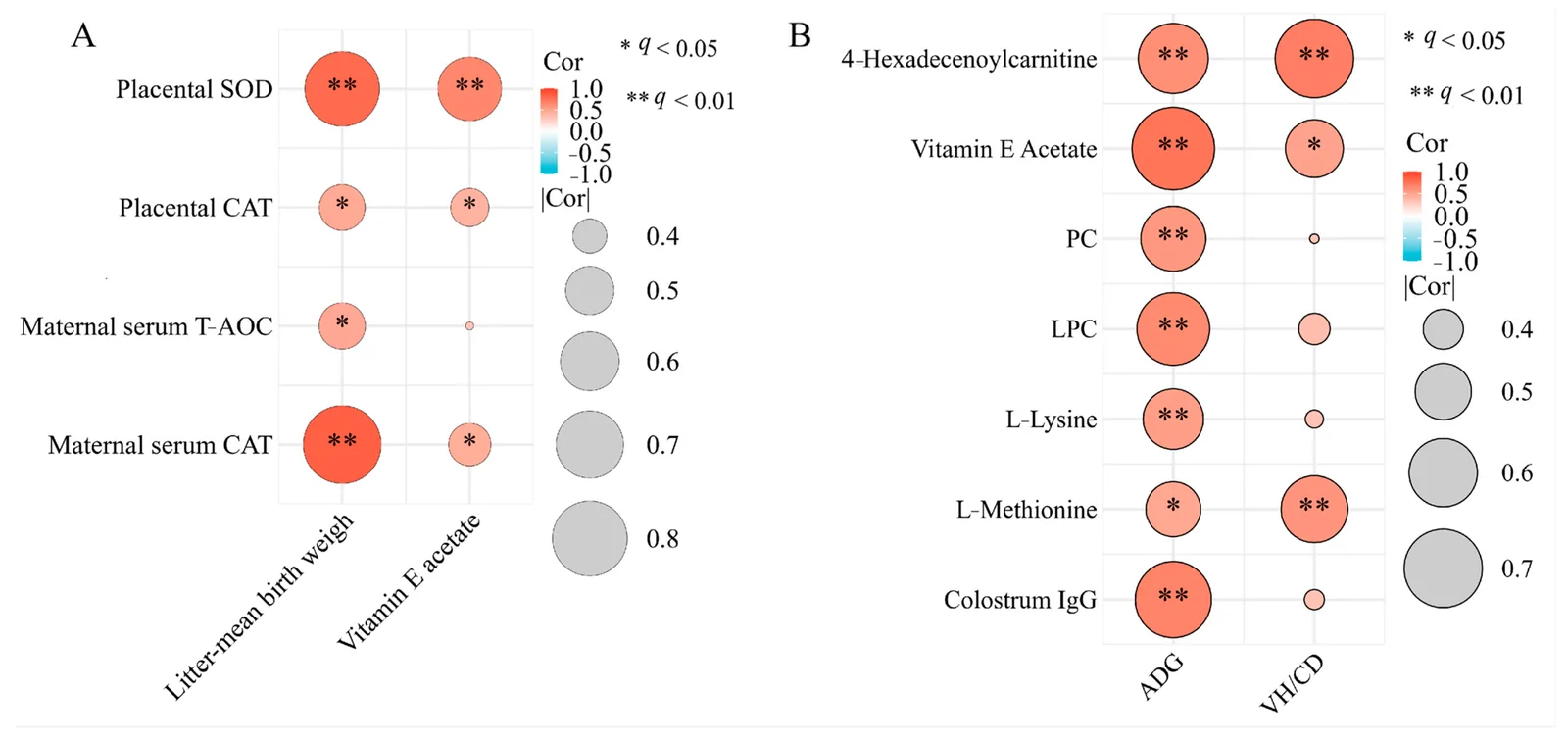
Partial Spearman rank correlation (controlling for treatment) among maternal antioxidant systems, colostrum composition, and offspring performance (*n* = 12 sows per group). (**A**) Correlations between maternal/placental antioxidant parameters and offspring birth weight or colostrum vitamin E acetate. (**B**) Correlations between colostrum components (metabolites and IgG) and offspring phenotypes (ADG and VH/CD). In both panels, Cor denotes partial Spearman’s rho (ρ). The color intensity indicates the direction of correlation (red: positive, blue: negative), and the ellipse size indicates the absolute magnitude (|ρ|). Asterisks indicate FDR4-corrected significance (* *q* < 0.05, ** *q* < 0.01).

Placental SOD and maternal serum CAT were positively correlated with litter-mean birth weight (*q* < 0.01), whereas placental CAT and maternal serum T-AOC showed weaker positive correlations (*q* < 0.05). Placental SOD, placental CAT, and maternal serum CAT were positively correlated with colostrum vitamin E acetate (*q* < 0.01, *q* < 0.05); maternal serum T-AOC showed no significant correlation (Figure 8A).

Colostrum 4-hexadecenoylcarnitine, vitamin E acetate, PC, LPC, L-lysine, L-methionine, and IgG were positively correlated with ADG (*q* < 0.05, *q* < 0.01). 4-Hexadecenoylcarnitine, vitamin E acetate, and L-methionine were positively correlated with VH/CD ratio (*q* < 0.05, *q* < 0.01); PC, LPC, L-lysine, and IgG showed no significant correlation with VH/CD ratio (Figure 8B).

## 4. Discussion

The core findings of this study indicate that CAE supplementation during late gestation and lactation resulted in altered maternal antioxidant status, modified colostrum composition, and shifted offspring phenotypes (litter-mean birth weight, ADG, intestinal morphology) and fecal microbiota structure. These observations support an integrative model of maternal nutritional programming. However, because supplementation spanned both late gestation and lactation without between-group cross-fostering, this design precludes isolating the relative contributions of gestational versus lactational exposure; nevertheless, random allocation permits causal attribution of the observed effects to CAE intervention, though the proposed mechanistic pathways require further validation.

Sow reproductive outcomes are a core determinant of economic efficiency in pig production [33], with piglet birth weight serving as a critical indicator of farm output. Among genetic, husbandry, and nutritional strategies, dietary intervention is considered a cost-effective and operationally feasible approach to improve reproductive efficiency [34]. In this study, CAE supplementation resulted in higher litter-mean birth weight than controls, consistent with the positive effects observed following peri-implantation CAE intervention [29], raising the possibility that CGA-rich extract may exert effects at multiple critical stages of the sow reproductive cycle. Compared with the periconception to early gestation effects reported by Zhang et al. [29], the present study extends the observation window to late gestation and lactation, representing an expansion of existing evidence.

This association may be partially linked to alterations in placental function. The placenta serves as the core interface for maternal–fetal exchange, where vascular development and antioxidant status are directly implicated in fetal nutrient supply [35]. The placenta is in a high metabolic state during gestation, and the antioxidant enzyme system (SOD and CAT) serves as a critical defense against oxidative stress-induced damage [36]; maintaining this system’s function is essential for protecting maternal–fetal exchange efficiency [37]. CGA, as the primary active component of CAE, has demonstrated free radical scavenging and anti-inflammatory activities [27,28], raising the possibility that it may alleviate oxidative stress-induced endothelial damage and thereby affect placental microvascular structure. In this study, the CAE group exhibited higher placental villous capillary density and placental antioxidant enzyme activity than controls, suggesting that redox status contributes to microvascular integrity in this model. However, whether higher capillary density translates to improved placental perfusion efficiency requires hemodynamic measurements for clarification. Moreover, while these placental differences are attributable to CAE intervention due to random allocation, they cannot be specifically attributed to the gestational component of exposure, as supplementation continued through lactation without a gestation-only exposure control.

Changes in maternal serum antioxidant indices may concurrently affect placental function, suggesting that placental antioxidant improvement may reflect the regulation of maternal systemic metabolic environment rather than an isolated local event. In the CAE group, maternal serum T-AOC and CAT activities, as well as placental SOD and CAT activities, were positively correlated with litter-mean birth weight, suggesting that maternal–fetal interface redox balance contributes to fetal growth outcomes; however, confirming that this pathway mediates the CAE effect requires mechanistic validation. Notably, the increase in placental SOD activity was selective and not accompanied by synchronous changes in maternal serum SOD, suggesting the existence of independent antioxidant regulatory mechanisms in the placenta whose biological significance warrants further exploration.

Beyond antioxidant parameters, CAE exposure was associated with concurrent changes in maternal immune indices: elevated serum immunoglobulins (IgA, IgG, IgM) and increased colostrum IgG concentration. Because the sow does not consume colostrum, this elevation is not attributable to local gastrointestinal exposure but may be associated with altered systemic maternal immune status. Colostrum IgG is predominantly derived from maternal circulation via neonatal Fc receptor (FcRn)-mediated transport, not de novo mammary synthesis [38,39], consistent with the classical mechanism of passive immune transfer. This pattern parallels other functional additive studies: silymarin administration significantly increased colostrum IgG, IgA, and IgM levels [40], and glycerol monolaurate feeding also increased IgA and IgG concentrations in colostrum and sow plasma [41], raising the possibility that specific dietary additives affect passive immune transfer to neonatal piglets through modulation of the maternal immune system. Elevated immunoglobulins may reflect enhanced maternal protein synthetic capacity, supported by three complementary mechanisms. First, CAE may modulate nutrient-sensing pathways such as mammalian target of rapamycin (mTOR) and AMP-activated protein kinase (AMPK), which regulate global protein anabolism, as reported for other polyphenolic compounds [42], thereby providing the translational machinery for increased antibody output. At the cellular level, CAE and its metabolites may act through oral routes on gut-associated lymphoid tissue and systemic secondary lymphoid organs, promoting B cell activation and differentiation into plasma cells, thereby expanding the immunoglobulin-producing cell pool. Complementing this expansion, the antioxidant and anti-inflammatory properties of CAE [27,28] may alleviate oxidative stress-induced immune cell damage, indirectly protecting or enhancing immunoglobulin synthesis. The relative contribution of each mechanism is unknown; whether enhanced protein synthesis specifically targets antibody-producing cells or reflects broader maternal metabolic reprogramming also remains undetermined. Distinguishing among these mechanisms and clarifying the specificity of protein synthetic enhancement will require in vitro lymphocyte or hepatocyte culture models, as well as in vivo intervention studies with temporal dissection of gestational versus lactational exposure. These mechanistic hypotheses find tentative observational parallels in the present study: despite equivalent colostrum intake, CAE piglets exhibited higher ADG, and the significant positive correlation between colostrum IgG and piglet ADG is consistent with a potential role of colostrum quality in early growth variation. However, whether colostrum IgG mediates the observed growth difference requires direct intervention.

Colostrum compositional alterations extended beyond immunoglobulins. Metabolomic analysis further revealed multi-level metabolic remodeling; however, untargeted metabolomics reflects static differences in metabolite abundance rather than distinguishing between enhanced synthesis, altered utilization, or passive turnover, nor does it infer metabolic flux. Therefore, functional interpretations of specific metabolites should be considered as mechanistic hypotheses requiring validation. Previous studies have demonstrated that nutritional interventions during late gestation and lactation in sows can significantly alter the colostrum metabolome and affect piglet growth [43,44,45]. The overall direction of metabolomic changes was compatible with the metabolic reprogramming characteristics of the perinatal-to-lactation transition [46], manifesting as extensive changes across multiple metabolite classes. Among these, vitamin E acetate was significantly elevated in colostrum following CAE supplementation. As the primary storage form of vitamin E, its increase was positively correlated with maternal serum CAT and placental SOD and CAT activities, providing observational evidence for systematic associations within the “maternal-placental-mammary” redox transfer and suggesting that oxidative reserves may be coordinately allocated among maternal tissues. Previous studies have also shown that high-dose vitamin E supplementation during late gestation and lactation can improve piglet humoral immunity and redox status [47,48]. The phenolic metabolite 4-hydroxyacetophenone was also increased, possessing hydrogen atom transfer potential, though its actual antioxidant contribution in colostrum remains unclear. The elevation of these two metabolites was coincident with the enhanced antioxidant capacity observed in CAE group piglets, suggesting that colostrum antioxidant components may be involved in the transfer of maternal oxidative protection to offspring.

Furthermore, multiple amino acids (such as lysine, methionine, and tyrosine) were significantly upregulated, consistent with previous results regarding dietary protein or specific amino acid regulation of free amino acid pools in milk [49]. Amino acids serve not only as precursors for protein synthesis but also may participate in neonatal intestinal growth and muscle development through activation of the mTOR pathway. The increase in specific acylcarnitines and fatty acid amide metabolites suggests that maternal nutritional intervention may enhance fatty acid oxidation energy supply and signaling potential in milk, a phenomenon previously reported in colostrum from sows fed probiotics or specific energy ratio diets [43,50]. Acylcarnitine abundance changes were consistent with enhanced maternal fatty acid mobilization during the perinatal period [51], while phospholipid profile remodeling may be related to secretory cell membrane composition adjustments [43]; their causal significance requires clarification through isotope tracing or flux analysis.

Concurrently, pyrimidine metabolism intermediates (such as orotic acid and UMP) and biotin were decreased in colostrum, possibly reflecting metabolic flux redistribution from de novo pyrimidine synthesis toward other energy metabolic pathways under this nutritional intervention. Zhang et al. [52] reported that 5′-UMP in human milk promotes thermogenesis and mitochondrial biogenesis; the decrease in pyrimidine metabolism intermediates in this study forms an interesting contrast with this finding, possibly reflecting species-specific differences (human vs. pig) or different metabolic programming strategies by intervention type (nucleotide supplementation vs. polyphenol intervention). However, this contrast is merely literature-based speculation and cannot exclude the influence of detection method sensitivity or individual variation. Although decreased N-palmitoylaspartate suggests that the N-acyl amino acid signaling axis may participate in polyphenol-induced metabolic reprogramming, the function of this metabolite in colostrum remains an emerging field lacking direct functional validation, and no inference can be made that this signaling axis is suppressed.

These differences in the colostrum metabolic environment may be associated with differences in piglet gut microbiota colonization patterns. At the microbial level, CAE supplementation was associated with significant differences in fecal microbial community composition, characterized by the co-enrichment of taxa reported in the literature to possess fiber degradation and butyrate production potential: *Cryptobacteroides* possesses extensive polysaccharide utilization loci [53]; *Treponema* (members of *Spirochaetota*) participate in fiber and pectin degradation [54,55]; certain *Sodaliphilus* MAGs carry butyrate synthesis genes [56]; and members of *Oscillospiraceae* (including *Vescimonas* and *Angelakisella*) as well as *Clostridia* within *Firmicutes_A* are major butyrate-producing taxa in the gut [48,57,58]. The co-enrichment of these taxa is compatible with the hypothesis that dietary polyphenols can modulate gut microbiota to affect fermentation potential [59]. However, the inference of enhanced butyrate production capacity is entirely based on 16S rRNA taxonomic data and represents a hypothesis-generating conclusion. Microbial genes were not directly sequenced, actual short-chain fatty acid (SCFA) concentrations were not determined, and metagenomic functional annotation was not performed. Whether this metabolic potential is realized, and whether this potential contributes to epithelial energy, barrier function, or growth performance requires targeted metabolite detection (such as fecal SCFA quantification) and functional genomics validation.

On the other hand, taxa associated with pathological or dysbiotic states in other studies (*Fusobacteriota*, *Proteobacteria*, *Fusobacterium_A*, *Escherichia*, *Mitsuokella*, and *Megasphaera_A*) were present at lower levels in the CAE group, raising the possibility that community structure may shift toward a lower pathogenic pressure state. Specifically, porcine epidemic diarrhea virus (PEDV) infection has been reported to be associated with higher Fusobacterium abundance [60]; enterotoxigenic *E. coli* (ETEC) is a major threat to intestinal health in lactating piglets [61]; co-enrichment of *Mitsuokella* and *Megasphaera* has been documented in post-weaning diarrhea in piglets [62], and *Megasphaera* is overrepresented in both D-galactose-induced chronic oxidative stress piglet models [63] and inflammatory bowel disease (IBD) patient intestines [64]. However, genus-level 16S rRNA sequencing cannot distinguish between pathogenic and commensal strains; therefore, the lower *Escherichia* abundance in the CAE group should not be interpreted as confirmatory evidence of reduced pathogen load. Moreover, the observed associations derive from a healthy sow population, and direct extrapolation to disease states is inappropriate.

At the community structure level, alpha diversity indices showed no significant differences between CAE and control groups, indicating comparable species richness and evenness. However, distinct clustering in beta diversity analysis revealed that maternal dietary CAE intervention substantially restructured offspring gut microbial community composition. This dissociation between alpha and beta diversity suggests that CAE selectively enriches specific functional taxa and reshapes microbial interaction networks, rather than simply altering total species number. This interpretation is consistent with LEfSe analysis showing co-enrichment of functional taxa (fiber degraders and butyrate producers) and synchronous reduction in pathology-related taxa, further supporting that CAE reshapes gut microecology through functional remodeling rather than diversity enhancement. However, given that these inferences derive from 16S rRNA taxonomic data alone, confirmation of strain-level identity and metabolic potential requires metagenomic and metabolomic validation.

In contrast to the above pattern, *Lactobacillus* abundance was lower in the CAE group, reflecting the complexity of polyphenol–lactobacillus interactions. A human study showed that habitual red wine drinkers (approximately 100 mL/day) had lower fecal *Lactobacillus* abundance than controls [65], raising the possibility that dietary polyphenols may inhibit lactobacillus colonization in specific host intestinal environments; conversely, vinegar polyphenol extract was associated with higher *Lactobacillus* and *Bifidobacterium* abundance in a diabetic mouse model [66], and tea polyphenols have also been reported to affect gut microbiota balance through selective enrichment of *Lactobacillus* and *Bifidobacterium* [59]. This indicates that the association between CAE and *Lactobacillus* abundance may be related to specific polyphenol components, host background, or intestinal microenvironment, rather than representing a simple unidirectional effect. Whether lower *Lactobacillus* abundance is associated with adverse consequences and whether this phenomenon represents a long-term adaptive response awaits clarification through longitudinal tracking and functional validation.

Integrating these observations, this study proposes an integrative framework for perinatal maternal nutritional programming: CAE exposure resulted in altered maternal antioxidant status, concurrent changes in colostrum immune protection and metabolic support, and shifts in early piglet gut microbiota colonization patterns, which collectively contributed to observed differences in intestinal morphology and growth performance. This framework extends prior evidence from early gestation [29] to late gestation and lactation, consistent with coordinated maternal–offspring physiological changes during the peripartum period. The following technical and measurement limitations should be considered when interpreting these findings. A critical design constraint is that CAE was continuously supplemented through late gestation and lactation without between-group cross-fostering, precluding independent attribution of effects to specific time windows. Technically, untargeted metabolomics reflects static abundance differences without metabolic flux information, and 16S rRNA sequencing lacks strain-level resolution for discriminating pathogenic from commensal strains. Functional inferences therefore rely on literature associations rather than direct genetic evidence, and fecal SCFA concentrations were not determined. At the measurement level, CAE was characterized only by CGA content, leaving other bioactive compounds unanalyzed; observed effects should be attributed to the CAE preparation as a whole. Placental morphological assessment sampled only one placenta per sow, without recording corresponding piglet characteristics (birth weight, sex, birth order) or placental weight. Consequently, these data cannot support inferences about individual fetal-placental units or within-litter variation, and should be interpreted as a single litter-level observation. Residual feed was not weighed, preventing precise determination of actual feed intake, which may introduce residual confounding.

To address these limitations, split-treatment designs isolating gestational versus lactational CAE exposure would disentangle time-specific contributions. Integration of metagenomics, metabolomics, and proteomics with in vitro functional assays would move beyond taxonomic and static metabolomic inference. Targeted supplementation trials of key metabolites could isolate their specific contributions from the CAE matrix. Precise individual feed intake measurement and sequential body condition monitoring would control nutritional confounding. These comprehensive efforts will contribute to establishing whether the observed effects represent causal pathways or secondary consequences of maternal metabolic remodeling.

## 5. Conclusions

CAE supplementation during late gestation and lactation was associated with altered maternal health, modified colostrum composition, and changes in piglet growth, intestinal morphology, and fecal microbiota composition. These findings identify late gestation and lactation as a window during which CAE exposure is associated with maternal and offspring outcomes, extending beyond early gestation effects previously reported. However, the relative contributions of gestational versus lactational exposure cannot be determined; while randomization permits causal attribution of treatment effects to CAE, the specific causal pathways linking CAE to maternal and offspring outcomes remain to be established through split-treatment designs and functional assays.

## Figures and Tables

**Table 1:** Ingredient compositions and nutrient levels of the sow basal diet.

Ingredient Composition (%)	Basal Diet (Gestation)	Basal Diet (Lactation)
Corn	53.60	61.60
Soybean Meal	17.50	23.50
Wheat Bran	12.00	4.00
Soybean Hulls	5	0.00
Rice Bran Meal	8.00	0.00
Fish Meal	0.00	2.00
Soybean Oil	0.00	3.00
Glucose	0.00	2.00
Limestone	1.00	1.30
Dicalcium Phosphate	1.20	1.20
Sodium Chloride	0.50	0.50
L-Lysine hydrochloride (98%)	0.15	0.15
L-Threonine (98.5%)	0.08	0.02
L-Valine (98.5%)	0.00	0.06
L-Tryptophan (98%)	0.00	0.02
Magnesium Oxide	0.15	0.00
Sodium Bicarbonate	0.20	0.00
Choline Chloride (50%)	0.07	0.10
Premix ^1^	0.55	0.55
Total	100	100
Nutrient levels ^2^		
Crude Protein (CP, %)	14.82	17.31
Crude Fat (EE, %)	3.98	6.08
Crude Fiber (CF, %)	6.17	3.29
Calcium (Ca, %)	0.80	0.96
Total Phosphorus (P, %)	0.69	0.65
Total Lysine (%)	0.86	1.02
Net Energy (NE, MJ/kg)	8.65	10.44

^1^ Premix (per kg diet): Fe 120 mg, Cu 20 mg, Mn 60 mg, Zn 120 mg, Se 0.3 mg, I 0.5 mg, VA 10,000 IU, VD_3_ 2000 IU, VE 60 IU, VK_3_ 5.0 mg, VB_1_ 5.0 mg, VB_2_ 10.0 mg, VB_6_ 6.0 mg, VB_12_ 50 μg, niacin 40 mg, pantothenic acid 20 mg, folic acid 2.0 mg, biotin 0.2 mg. ^2^ CP, EE, CF, Ca, and P were determined by analysis; the other values were calculated.

## Data Availability

The 16S rRNA gene sequencing data have been deposited in the NCBI Sequence Read Archive (SRA) under BioProject ID PRJNA1456343 (https://www.ncbi.nlm.nih.gov/bioproject/PRJNA1456343, accessed on 12 September 2026). Additional datasets generated during this study are available from the corresponding authors upon reasonable request.
